# Supplementary material for: Do popular apps have issues regarding energy efficiency?
Source: PeerJ Comput Sci. 2024 Feb 29;10:e1891. doi: 10.7717/peerj-cs.1891 (PMC10909214; doi:10.7717/peerj-cs.1891)
Supplement: Supplemental Information 1 — Code for getting reviews [file peerj-cs-10-1891-s001.zip › AppReviewCollector/TermProject_DotNet/TermProject_DotNet/Views/Shared/_Layout.cshtml]

@ViewData["Title"] - TermProject\_DotNet


TermProject\_DotNet


- Home

@RenderBody()

© 2022 - TermProject\_DotNet

@await RenderSectionAsync("Scripts", required: false)
