# Supplementary figures and images for: Do popular apps have issues regarding energy efficiency?
Source: PeerJ Comput Sci. 2024 Feb 29;10:e1891. doi: 10.7717/peerj-cs.1891 (PMC10909214; doi:10.7717/peerj-cs.1891)

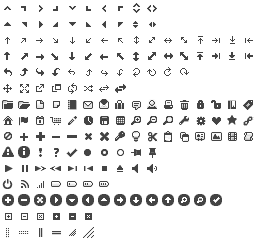

Supplement: Supplemental Information 1 — Code for getting reviews [file peerj-cs-10-1891-s001.zip › AppReviewCollector/TermProject_DotNet/TermProject_DotNet/wwwroot/lib/jqueryui/images/ui-icons_444444_256x240.png]

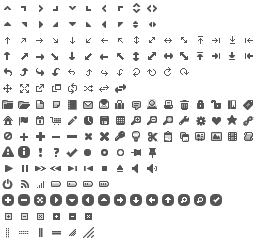

Supplement: Supplemental Information 1 — Code for getting reviews [file peerj-cs-10-1891-s001.zip › AppReviewCollector/TermProject_DotNet/TermProject_DotNet/wwwroot/lib/jqueryui/images/ui-icons_555555_256x240.png]

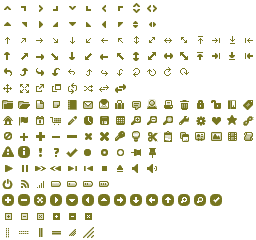

Supplement: Supplemental Information 1 — Code for getting reviews [file peerj-cs-10-1891-s001.zip › AppReviewCollector/TermProject_DotNet/TermProject_DotNet/wwwroot/lib/jqueryui/images/ui-icons_777620_256x240.png]

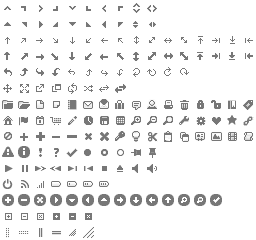

Supplement: Supplemental Information 1 — Code for getting reviews [file peerj-cs-10-1891-s001.zip › AppReviewCollector/TermProject_DotNet/TermProject_DotNet/wwwroot/lib/jqueryui/images/ui-icons_777777_256x240.png]

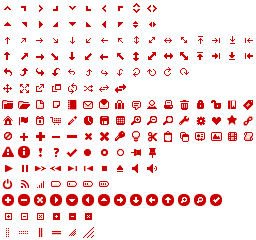

Supplement: Supplemental Information 1 — Code for getting reviews [file peerj-cs-10-1891-s001.zip › AppReviewCollector/TermProject_DotNet/TermProject_DotNet/wwwroot/lib/jqueryui/images/ui-icons_cc0000_256x240.png]

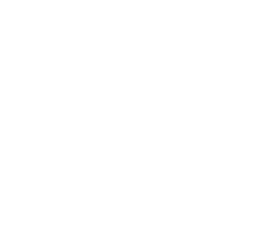

Supplement: Supplemental Information 1 — Code for getting reviews [file peerj-cs-10-1891-s001.zip › AppReviewCollector/TermProject_DotNet/TermProject_DotNet/wwwroot/lib/jqueryui/images/ui-icons_ffffff_256x240.png]

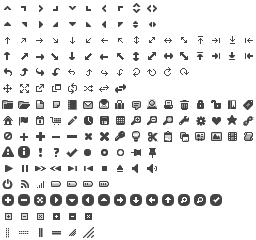

Supplement: Supplemental Information 1 — Code for getting reviews [file peerj-cs-10-1891-s001.zip › AppReviewCollector/TermProject_DotNet/TermProject_DotNet/wwwroot/lib/jqueryui/themes/base/images/ui-icons_444444_256x240.png]

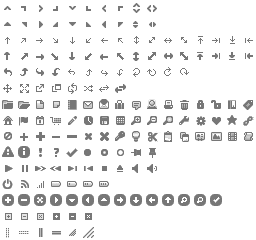

Supplement: Supplemental Information 1 — Code for getting reviews [file peerj-cs-10-1891-s001.zip › AppReviewCollector/TermProject_DotNet/TermProject_DotNet/wwwroot/lib/jqueryui/themes/base/images/ui-icons_777777_256x240.png]

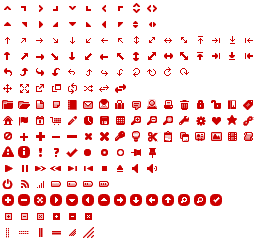

Supplement: Supplemental Information 1 — Code for getting reviews [file peerj-cs-10-1891-s001.zip › AppReviewCollector/TermProject_DotNet/TermProject_DotNet/wwwroot/lib/jqueryui/themes/base/images/ui-icons_cc0000_256x240.png]

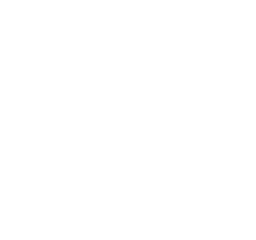

Supplement: Supplemental Information 1 — Code for getting reviews [file peerj-cs-10-1891-s001.zip › AppReviewCollector/TermProject_DotNet/TermProject_DotNet/wwwroot/lib/jqueryui/themes/base/images/ui-icons_ffffff_256x240.png]

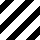

Supplement: Supplemental Information 1 — Code for getting reviews [file peerj-cs-10-1891-s001.zip › AppReviewCollector/TermProject_DotNet/TermProject_DotNet/wwwroot/lib/jqueryui/themes/black-tie/images/ui-bg_diagonals-thick_8_333333_40x40.png]

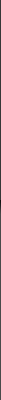

Supplement: Supplemental Information 1 — Code for getting reviews [file peerj-cs-10-1891-s001.zip › AppReviewCollector/TermProject_DotNet/TermProject_DotNet/wwwroot/lib/jqueryui/themes/black-tie/images/ui-bg_glass_40_111111_1x400.png]

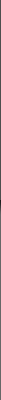

Supplement: Supplemental Information 1 — Code for getting reviews [file peerj-cs-10-1891-s001.zip › AppReviewCollector/TermProject_DotNet/TermProject_DotNet/wwwroot/lib/jqueryui/themes/black-tie/images/ui-bg_glass_55_1c1c1c_1x400.png]

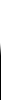

Supplement: Supplemental Information 1 — Code for getting reviews [file peerj-cs-10-1891-s001.zip › AppReviewCollector/TermProject_DotNet/TermProject_DotNet/wwwroot/lib/jqueryui/themes/black-tie/images/ui-bg_highlight-hard_100_f9f9f9_1x100.png]

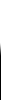

Supplement: Supplemental Information 1 — Code for getting reviews [file peerj-cs-10-1891-s001.zip › AppReviewCollector/TermProject_DotNet/TermProject_DotNet/wwwroot/lib/jqueryui/themes/black-tie/images/ui-bg_highlight-hard_40_aaaaaa_1x100.png]

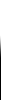

Supplement: Supplemental Information 1 — Code for getting reviews [file peerj-cs-10-1891-s001.zip › AppReviewCollector/TermProject_DotNet/TermProject_DotNet/wwwroot/lib/jqueryui/themes/black-tie/images/ui-bg_highlight-soft_50_aaaaaa_1x100.png]

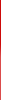

Supplement: Supplemental Information 1 — Code for getting reviews [file peerj-cs-10-1891-s001.zip › AppReviewCollector/TermProject_DotNet/TermProject_DotNet/wwwroot/lib/jqueryui/themes/black-tie/images/ui-bg_inset-hard_45_cd0a0a_1x100.png]

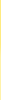

Supplement: Supplemental Information 1 — Code for getting reviews [file peerj-cs-10-1891-s001.zip › AppReviewCollector/TermProject_DotNet/TermProject_DotNet/wwwroot/lib/jqueryui/themes/black-tie/images/ui-bg_inset-hard_55_ffeb80_1x100.png]

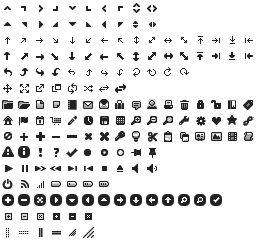

Supplement: Supplemental Information 1 — Code for getting reviews [file peerj-cs-10-1891-s001.zip › AppReviewCollector/TermProject_DotNet/TermProject_DotNet/wwwroot/lib/jqueryui/themes/black-tie/images/ui-icons_222222_256x240.png]

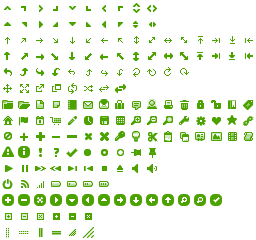

Supplement: Supplemental Information 1 — Code for getting reviews [file peerj-cs-10-1891-s001.zip › AppReviewCollector/TermProject_DotNet/TermProject_DotNet/wwwroot/lib/jqueryui/themes/black-tie/images/ui-icons_4ca300_256x240.png]

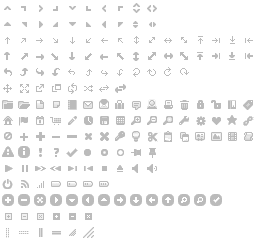

Supplement: Supplemental Information 1 — Code for getting reviews [file peerj-cs-10-1891-s001.zip › AppReviewCollector/TermProject_DotNet/TermProject_DotNet/wwwroot/lib/jqueryui/themes/black-tie/images/ui-icons_bbbbbb_256x240.png]

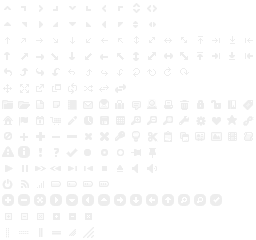

Supplement: Supplemental Information 1 — Code for getting reviews [file peerj-cs-10-1891-s001.zip › AppReviewCollector/TermProject_DotNet/TermProject_DotNet/wwwroot/lib/jqueryui/themes/black-tie/images/ui-icons_ededed_256x240.png]

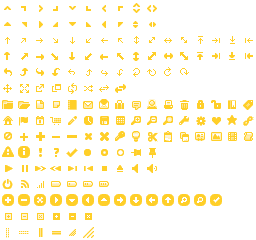

Supplement: Supplemental Information 1 — Code for getting reviews [file peerj-cs-10-1891-s001.zip › AppReviewCollector/TermProject_DotNet/TermProject_DotNet/wwwroot/lib/jqueryui/themes/black-tie/images/ui-icons_ffcf29_256x240.png]

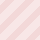

Supplement: Supplemental Information 1 — Code for getting reviews [file peerj-cs-10-1891-s001.zip › AppReviewCollector/TermProject_DotNet/TermProject_DotNet/wwwroot/lib/jqueryui/themes/blitzer/images/ui-bg_diagonals-thick_75_f3d8d8_40x40.png]

Supplement: Supplemental Information 1 — Code for getting reviews [file peerj-cs-10-1891-s001.zip › AppReviewCollector/TermProject_DotNet/TermProject_DotNet/wwwroot/lib/jqueryui/themes/blitzer/images/ui-bg_dots-small_65_a6a6a6_2x2.png]

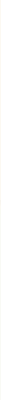

Supplement: Supplemental Information 1 — Code for getting reviews [file peerj-cs-10-1891-s001.zip › AppReviewCollector/TermProject_DotNet/TermProject_DotNet/wwwroot/lib/jqueryui/themes/blitzer/images/ui-bg_glass_55_fbf8ee_1x400.png]

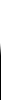

Supplement: Supplemental Information 1 — Code for getting reviews [file peerj-cs-10-1891-s001.zip › AppReviewCollector/TermProject_DotNet/TermProject_DotNet/wwwroot/lib/jqueryui/themes/blitzer/images/ui-bg_highlight-hard_100_eeeeee_1x100.png]

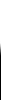

Supplement: Supplemental Information 1 — Code for getting reviews [file peerj-cs-10-1891-s001.zip › AppReviewCollector/TermProject_DotNet/TermProject_DotNet/wwwroot/lib/jqueryui/themes/blitzer/images/ui-bg_highlight-hard_100_f6f6f6_1x100.png]

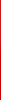

Supplement: Supplemental Information 1 — Code for getting reviews [file peerj-cs-10-1891-s001.zip › AppReviewCollector/TermProject_DotNet/TermProject_DotNet/wwwroot/lib/jqueryui/themes/blitzer/images/ui-bg_highlight-soft_15_cc0000_1x100.png]

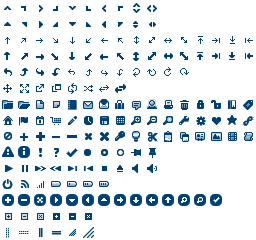

Supplement: Supplemental Information 1 — Code for getting reviews [file peerj-cs-10-1891-s001.zip › AppReviewCollector/TermProject_DotNet/TermProject_DotNet/wwwroot/lib/jqueryui/themes/blitzer/images/ui-icons_004276_256x240.png]

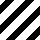

Supplement: Supplemental Information 1 — Code for getting reviews [file peerj-cs-10-1891-s001.zip › AppReviewCollector/TermProject_DotNet/TermProject_DotNet/wwwroot/lib/jqueryui/themes/cupertino/images/ui-bg_diagonals-thick_90_eeeeee_40x40.png]

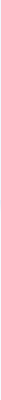

Supplement: Supplemental Information 1 — Code for getting reviews [file peerj-cs-10-1891-s001.zip › AppReviewCollector/TermProject_DotNet/TermProject_DotNet/wwwroot/lib/jqueryui/themes/cupertino/images/ui-bg_glass_100_e4f1fb_1x400.png]

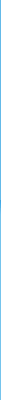

Supplement: Supplemental Information 1 — Code for getting reviews [file peerj-cs-10-1891-s001.zip › AppReviewCollector/TermProject_DotNet/TermProject_DotNet/wwwroot/lib/jqueryui/themes/cupertino/images/ui-bg_glass_50_3baae3_1x400.png]

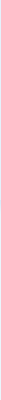

Supplement: Supplemental Information 1 — Code for getting reviews [file peerj-cs-10-1891-s001.zip › AppReviewCollector/TermProject_DotNet/TermProject_DotNet/wwwroot/lib/jqueryui/themes/cupertino/images/ui-bg_glass_80_d7ebf9_1x400.png]

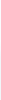

Supplement: Supplemental Information 1 — Code for getting reviews [file peerj-cs-10-1891-s001.zip › AppReviewCollector/TermProject_DotNet/TermProject_DotNet/wwwroot/lib/jqueryui/themes/cupertino/images/ui-bg_highlight-hard_100_f2f5f7_1x100.png]

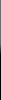

Supplement: Supplemental Information 1 — Code for getting reviews [file peerj-cs-10-1891-s001.zip › AppReviewCollector/TermProject_DotNet/TermProject_DotNet/wwwroot/lib/jqueryui/themes/cupertino/images/ui-bg_highlight-hard_70_000000_1x100.png]

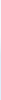

Supplement: Supplemental Information 1 — Code for getting reviews [file peerj-cs-10-1891-s001.zip › AppReviewCollector/TermProject_DotNet/TermProject_DotNet/wwwroot/lib/jqueryui/themes/cupertino/images/ui-bg_highlight-soft_100_deedf7_1x100.png]

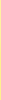

Supplement: Supplemental Information 1 — Code for getting reviews [file peerj-cs-10-1891-s001.zip › AppReviewCollector/TermProject_DotNet/TermProject_DotNet/wwwroot/lib/jqueryui/themes/cupertino/images/ui-bg_highlight-soft_25_ffef8f_1x100.png]

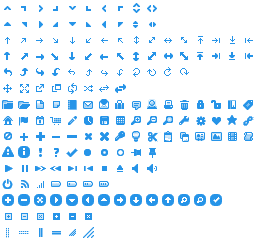

Supplement: Supplemental Information 1 — Code for getting reviews [file peerj-cs-10-1891-s001.zip › AppReviewCollector/TermProject_DotNet/TermProject_DotNet/wwwroot/lib/jqueryui/themes/cupertino/images/ui-icons_2694e8_256x240.png]

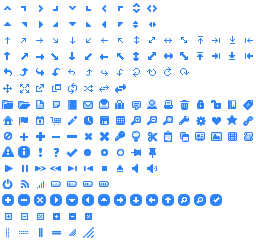

Supplement: Supplemental Information 1 — Code for getting reviews [file peerj-cs-10-1891-s001.zip › AppReviewCollector/TermProject_DotNet/TermProject_DotNet/wwwroot/lib/jqueryui/themes/cupertino/images/ui-icons_2e83ff_256x240.png]

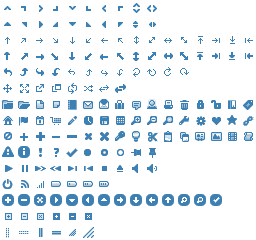

Supplement: Supplemental Information 1 — Code for getting reviews [file peerj-cs-10-1891-s001.zip › AppReviewCollector/TermProject_DotNet/TermProject_DotNet/wwwroot/lib/jqueryui/themes/cupertino/images/ui-icons_3d80b3_256x240.png]

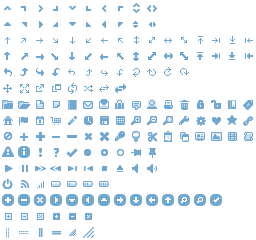

Supplement: Supplemental Information 1 — Code for getting reviews [file peerj-cs-10-1891-s001.zip › AppReviewCollector/TermProject_DotNet/TermProject_DotNet/wwwroot/lib/jqueryui/themes/cupertino/images/ui-icons_72a7cf_256x240.png]
